# The Renaissance of CDK Inhibitors in Breast Cancer Therapy: An Update on Clinical Trials and Therapy Resistance

**DOI:** 10.3390/cancers14215388

**Published:** 2022-11-01

**Authors:** Mary Abdelmalak, Rajanbir Singh, Mohammed Anwer, Pavel Ivanchenko, Amritdeep Randhawa, Myra Ahmed, Anthony W. Ashton, Yanming Du, Xuanmao Jiao, Richard Pestell

**Affiliations:** 1 Pennsylvania Cancer and Regenerative Medicine Research Center, Baruch S. Blumberg Institute, 3805 Old Easton Road, Doylestown, PA 18902, USA; 2 Xavier University School of Medicine, #23, Santa Helenastraat, Oranjestad, Aruba; 3 Lankenau Institute for Medical Research Philadelphia, 100 East Lancaster Ave., Wynnewood, PA 19069, USA; 4 The Wistar Cancer Center, Philadelphia, PA 19107, USA

**Keywords:** abemaciclib, advanced breast cancer, CDK4/6 inhibitor, hormone receptor-positive, palbociclib, ribociclib

## Abstract

**Simple Summary:**

Cyclin-dependent kinase inhibitors (palbociclib (Ibrance), ribociclib (Kisqali), and abemaciclib (Verzenio)), targeting aberrant cell-cycle activity have been evaluated extensively in clinical trials. Significant delays in progression free survival and overall survival are now documented with each agent in estrogen receptor positive and human epidermal growth factor receptor two negative advanced breast cancer including luminal B breast cancer. Therapy resistance, driven by chromosomal instability, results in genomic rearrangements, activation of cell-cycle components (cyclin E/cdk2 in Rb^−^ tumors, cyclin D1 in growth factor activated pathways), and the immune response. Molecular analysis of therapy resistant tumors may provide the rational basis for new therapies (brivanib, CYC065, WEE1 kinase and other inhibitors). Luminal B breast cancer is enriched for cyclin D1 overexpression and the chromosomal instability gene signature. The molecular mechanisms governing chromosomal instability in luminal B breast cancer remain poorly understood. Co-targeting of chromosomal instability may potentially reduce the prevalent escape mechanisms that reduce the effectiveness of cyclin-dependent kinase inhibitors.

**Abstract:**

Cyclin-dependent kinases (CDKs) govern cell-cycle checkpoint transitions necessary for cancer cell proliferation. Recent developments have illustrated nuanced important differences between mono CDK inhibitor (CDKI) treatment and the combination therapies of breast cancers. The CDKIs that are currently FDA-approved for breast cancer therapy are oral agents that selectively inhibit CDK4 and CDK6, include palbociclib (Ibrance), ribociclib (Kisqali), and abemaciclib (Verzenio). CDKI therapy is effective in hormone receptor positive (HR^+^), and human epidermal growth factor receptor two negative (HER2^−^) advanced breast cancers (ABC) malignancies, but remains susceptible due to estrogen and progesterone receptor overexpression. Adding a CDK4/6I to endocrine therapy increases efficacy and delays disease progression. Given the side effects of CDKI, identifying potential new treatments to enhance CDKI effectiveness is essential. Recent long-term studies with Palbociclib, including the PALLAS and PENELOPE B, which failed to meet their primary endpoints of influencing progression-free survival, suggest a deeper mechanistic understanding of cyclin/CDK functions is required. The impact of CDKI on the anti-tumor immune response represents an area of great promise. CDKI therapy resistance that arises provides the opportunity for specific types of new therapies currently in clinical trials.

## 1. Introduction

Despite the extensive use of anti-hormonal endocrine therapy and chemotherapy, therapy resistance and long-term side effects have led to the introduction of alternative treatments targeting the cell-cycle machinery. The essential role of CDKs in promoting cell cycle progression (through phosphorylation of Retinoblastoma (Rb)) established CDK4/6Is as a selective target therapy to influence outcomes in breast cancer, especially therapy-resistant breast cancer [1]. In clinical practice, the most commonly used CDK4/6Is include palbociclib, ribociclib, and abemaciclib for HR^+^ advanced breast malignancies. Developments of these agents derive from carefully controlled research trials. Three FDA-approved CDK4/6 inhibitors are palbociclib, ribociclib, and abemaciclib. Ribociclib is very similar to palbociclib in structure, but abemaciclib is different. In vitro studies indicated that palbociclib has an almost equivalent inhibition effect on CDK4 and CDK6, while abemaciclib and ribociclib are more potent against CDK4 than CDK6 [2,3,4]. Other inhibitors of CDK including CDK5 are in development and have been reviewed elsewhere [5]. The current review focuses on CDK4/6 inhibitors, to provide a clinical update noting the recent disappointments and a synopsis of the mechanisms governing therapy resistance and potential alternative approaches for the treatment of such resistance.

Several clinical trials formed a platform upon which the clinical utility of CDKI are based, including the PALOMA (Palbociclib Ongoing Trials in the Management of Breast Cancer) studies, showed significant results for palbociclib use, whereas the “Mammary Oncology assessment of LEE011’s (ribociclib’s) Efficacy and Safety” (MONALEESA) trials have proven ribociclib therapy as a viable treatment option. In addition, the MONARCH research studies have revealed improved survival outcomes on abemaciclib therapy in ABC patients.

## 2. Breast Cancer and Existing Targeted Therapy

Only to certain dermatologic malignancies, breast cancer leads to female mortality in the United States [6]. Breast cancer may be characterized based on coding [7] or non-coding genome [8]. The three most common molecular subtypes of breast cancers based on the coding genome are HR^+^ malignancies (estrogen receptor-positive (ER^+^) and progesterone receptor positive (PR^+^) breast cancers) [9], HER2^+^, and triple-negative breast cancer (TNBC) malignancies. HR^+^ breast cancer cells retain sensitivity to estrogen/progesterone-blocking endocrine therapy. HR^−^ breast cancer types are without a rational basis for endocrine treatments. HER2^+^ breast cancer cells display amplified expression of HER2 receptors that is targeted by antibodies, including the humanized monoclonal antibody trastuzumab (Herceptin) [10,11,12,13]. In contrast, TNBC malignancies which have the worst prognosis, do not express estrogen receptor (ER), progesterone receptor (PR), or HER2/neu, and are now being assessed for new targeted therapies to PARP, Trop2, CCR5 [14], DNMT1, VEGF and immune checkpoints [15,16].

Luminal A breast cancers express hormone receptors (ER^+^ and PR^+/−^) but lack expression of HER2 and have low levels of Ki-67 protein (a marker of proliferation). These subtypes grow slowly, are low-grade, with the best prognosis, and are most likely to benefit from hormone therapy but may not benefit from chemotherapy [17], and side effects from chemotherapy may outweigh the value of chemotherapy in this population [18]. Luminal B breast tumors are ER^+^ and PR^+/−^ and HER2^+/−^, with increased levels of Ki-67. Such subtypes grow faster with a slightly worse prognosis than Luminal A, with the best response to endocrine therapy, chemotherapy, and targeted anti-HER2 therapy in HER2^+^ patients [17,19,20]. HER2^+^ tumors are HER2-enriched, PR^−^/HR^−^, and tend to grow faster and benefit most from targeted therapies against HER2. Basal-like breast cancer, often referred to as TNBC due to its lack of ER, PR, and HER2 expression, is the most common subtype amongst patients with breast cancer gene 1 (BRCA1) mutations (early-onset) and African American women [21].

Factors contributing to breast cancer development include strong family history, BRCA1 and breast cancer gene 2 (BRCA2) gene mutations, a history of atypical hyperplasia, alcohol intake, and increased age. Ethnicity also plays a role, with TNBC being most prevalent in African American women. In contrast, Caucasian women are more susceptible to other breast cancer subtypes, particularly infiltrating ductal carcinoma, lobular carcinoma, and tubular adenocarcinoma [15]. In addition, higher estrogen exposure throughout a patient’s lifetime (i.e., early menstruation, late menopause, nulliparity, postmenopausal obesity, the elevated total number of menstrual cycles, absence of breastfeeding, later age of first pregnancy, alcohol intake, Klinefelter syndrome in men, etc.) can increase the risk of breast cancer [22].

**Figure 1:**
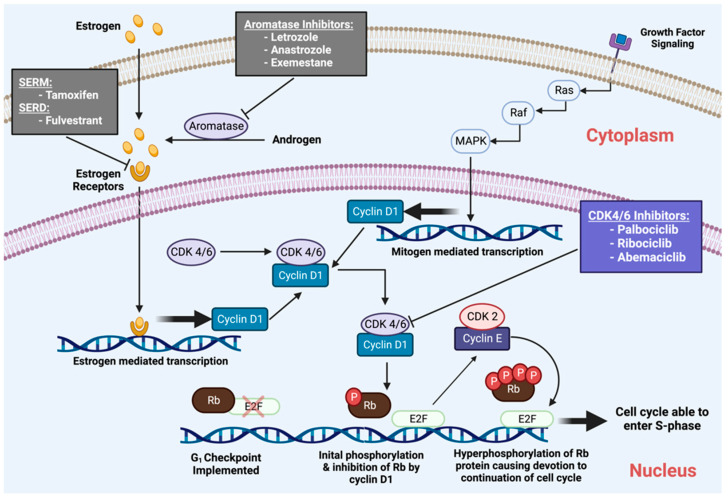
CDK in Breast Cancer therapy.

Anti-estrogen treatments include aromatase inhibitors, such as letrozole, and estrogen receptor antagonists, such as fulvestrant. However, a consequence of these long-term therapies is acquired resistance. Therefore, additional agents are required to attenuate the mechanisms of resistance and its associated rise in breast cancer mortality rates. To prevent therapeutic resistance, long-term therapies for breast malignancies must be consistent, stable, and, most importantly, selective to the target cancer cells [23]. The specificity of the CDK4/6Is, and evidence for increased CDK activity in HR^+^ breast cancers suggested the potential utility of CDK4/6Is in combination with endocrine therapy for therapy-specific breast cancer subtypes [24,25] (Figure 1).

## 3. The Regulatory Cyclin D Subunit Are Frequently Amplified in Human Breast Cancer

The cyclin regulatory subunit together with the catalytic CDK subunit generates a holoenzyme that phosphorylates gatekeeper proteins coordinating cell cycle progression [26]. The cell cycle is divided into phases, controlled by checkpoint transitions that proceed in an orderly and precise manner to ensure cellular growth and proliferation. Cyclin/CDK complexes must be activated and inactivated at appropriate times to ensure carefully timed progress through the cell cycle [26]. The holoenzymes are serine and threonine kinases that regulate cell cycle progression via selective phosphorylation of target substrates [27]. CDK4, and CDK6, together, with their cyclin-D regulatory subunits, promote the G1/S phase progression of the cell cycle [28]. The cyclin-CDK holoenzyme complexes phosphorylate the Rb protein to release elongation factor two (E2F) and promote DNA synthesis [28].

**Figure 2:**
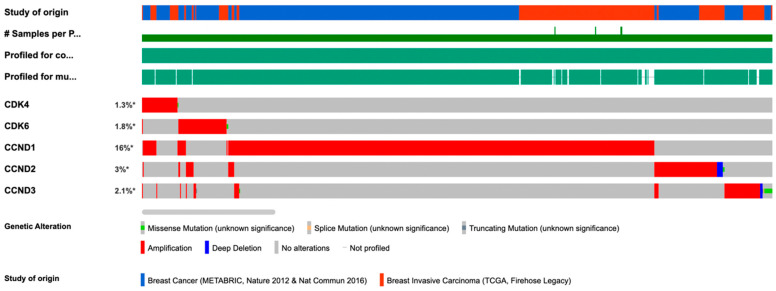
Infrequent CDK4/6 amplification in Breast Cancer therapy. Analysis of 3617 breast cancer samples from CBIOPORTSAL, combining the METABRIC and TCGA (Firehose Legacy data) showed amplification of CDK4 is a rare event (1.3%), co-occurring with cyclin D1 amplification. Frequent amplification of the regulatory subunit of the cyclin D/CDK complexes (cyclin D1, *CCND1*) occurs in human breast cancer. # number of samples. * percentage of patients in the population with the particular genetic abnormality.

The *CCND1* gene encodes cyclin D1 is frequently amplified in human breast cancers [2]. Analysis of 3617 samples, combining the METABRIC and TCGA (Firehose Legacy data) showed amplification of CDK4 is a rare event (1.3%) co-occurring with cyclin D1 amplification (Figure 2). Cyclin D1 protein abundance is increased as a consequence of overexpression, gene amplification, transcriptional induction or post-transcriptional induction, in >50% of breast cancers [2].

**Figure 3:**
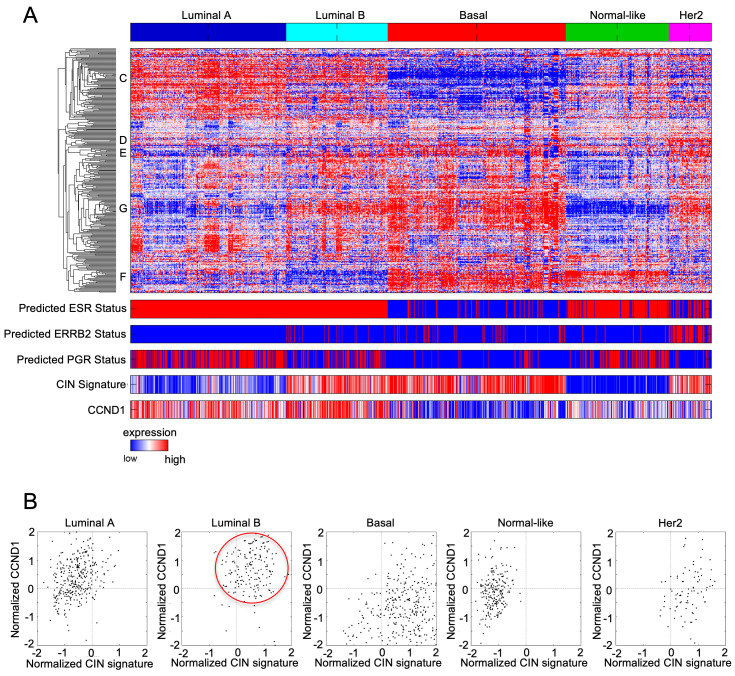
Chromosomal instability signature and cyclin D1 expression in breast cancer subtypes. (**A**) Heatmap of breast cancer microarray datasets assigned to the breast cancer coding genome expression subtypes. The predicted ESR1, epidermal growth factor receptor (ERBB2), and progesterone receptor (PGR) statuses are shown together with a chromosomal instability gene signature (CIN) signature score and *CCND1* expression level across the 5 subtypes. The CIN signature score, and cyclin D1 expression level are outlined for the luminal B subtype. (**B**) *CCND1* transcript level plotted vs. CIN signature expression level show a correlation between high CIN score and high cyclin D1 expression in luminal B subtype specific (red circle).

Cyclin D1 is overexpressed primarily in luminal breast cancer (luminal A and luminal B) associated with ERα^+^ breast cancer (Figure 3A). Consistent with the model in which cyclin D1 induces chromosomal instability, increased cyclin D1 correlates with the expression of chromosomal instability signature (Figure 3B). In tissue culture, CDKI reduces RB protein phosphorylation, reducing the release E2F from binding to Rb and G1 cell cycle arrest [29,30,31]. In addition, CDKIs have additional anticancer effects in breast cancer, including enhancing cancer cell immunogenicity and promoting cellular senescence [32].

## 4. CDK Inhibitors

Early efforts to produce CDKIs resulted in relatively non-selective targeting of several CDKs. However, the current generation CDKIs more specifically target CDK4 and CDK6, allowing for better toleration and reduced toxicity. The current FDA-approved CDKIs decrease phosphorylation of the RB tumor suppressor, promoting cell cycle arrest at the G1/S transition checkpoint. Current NCCN Guideline recommendations for metastatic HR^+^/HER2^−^, breast cancer include the addition of CDK4/6Is with hormonal therapy (letrozole, fulvestrant) in postmenopausal and for premenopausal patients as a preferred first-line treatment (Table 1, [33]). Palbociclib is a highly specific inhibitor of cyclin-dependent kinase 4 (Cdk4) (IC50, 0.011 µM) and Cdk6 (IC50, 0.016 µM).

**Palbociclib**. Palbociclib is a specific CDK4/6I. Palbociclib-responsive breast tumors are ER^+^, Rb^+^ with cyclin D1 overexpression. Palbociclib is best used with an aromatase inhibitor, frequently used in postmenopausal women and men who have not had prior hormonal therapy [44]. The aromatase inhibitors regularly used in combination therapy include Arimidex/anastrozole, Aromasin/exemestane, and Femara/letrozole. However, in all patients with previous hormonal therapy, palbociclib is best combined with the ER antagonist Faslodex/fulvestrant [45,46,47,48,49,50,51,52]. Female patients on this latter regimen must be on an LHRH (luteinizing hormone releasing-hormone) agonist to suppress ovarian function as the palbociclib/ER antagonist combination therapy results in continuous stimulation of the hypothalamic–pituitary–ovarian axis feedback loop, thereby inhibiting ovarian estrogen production, allowing tumor growth suppression [53].

PALOMA-1 was the first of many trials to analyze the therapeutic effects of combination therapy with palbociclib and letrozole in advanced breast cancer (ABC). The PALOMA-1 trial randomized postmenopausal women with ER^+^/HER2^−^ ABC to letrozole alone (2.5 milligrams (mg) daily) or in combination with palbociclib (125 mg daily). Palbociclib augmented progression-free survival (PFS) in the patients taking letrozole combination therapy, (Table 2 [54]) resulting in FDA approval (April 2013 [37]).

PALOMA-2, a phase III double-blind research study, was performed to further evaluate the outcomes of PALOMA-1. PALOMA-2 employed the exact dosage and scheduling of Palbociclib as PALOMA-1. Palbociclib treatment resulted in the extension of PFS compared to the placebo-controlled group, as shown in Table 2. Due to the greater number of patients, there was a more significant benefit observed in all breast cancer patient subgroups in PALOMA-2. This includes patients with lobular carcinoma and those who developed metastasis within 12 months of diagnosis, considered advanced breast cancer [55], resulting in FDA accelerated approval for palbociclib in February 2015.

The double-blinded phase III PALOMA-3 trial sought to assess the potential for combination therapy with palbociclib and the most common selective estrogen receptor degraders, fulvestrant. The patients treated were ER^+^, HER2^−^ breast cancers. The palbociclib group experienced extension to PFS and OS compared to placebo (Table 2) [56]. In addition, although palbociclib toxicity was frequent, improvement in global quality of life was noted [56,57,58].

The “Palbociclib Collaborative Adjuvant Study” (PALLAS) was initiated in 2015. to determine disease-free survival (iDFS) in patients who received palbociclib with an endocrine therapy versus a standalone endocrine therapy. Ultimately, 5761 patients were randomly assigned to either treatment group, with 2884 receiving palbociclib and endocrine therapy, and 2877 received only endocrine therapy, with each group receiving treatment for at least 5 years. The palbociclib was dosed at 125 mg orally given once daily for 21 days, followed by a 7-day break from treatment in a cycle of 28 days, and this continued for 2 years, along with a standard endocrine adjuvant for 5 years. At the median follow-up period of 31 months, the iDFS were compared for both treatment groups. The results showed that iDFS occurred at a rate of 8.8% in the patients receiving palbociclib plus the endocrine adjuvant and at a rate of 9.1% in patients just receiving the standard endocrine therapy on its own. Palbociclib added to an adjuvant endocrine therapy did not significantly improve compared to endocrine therapy on its own [59]. Palbociclib is not recommended in the adjuvant setting of stage II/III h ERα+, HER2^−^ breast cancer because the addition of Palbociclib to standard endocrine therapy (ET) did not improve outcomes. The molecular mechanisms governing this outcome is investigated through assessment of the Trans-PALLAS program samples.

The PENELOPE-B trials were conducted to assess the efficacy of combining palbociclib with adjuvant chemotherapy in breast cancer treatment outcomes. The PENELOPE-B trials randomly assigned patients to either 13 cycles for 4 weeks at a time of palbociclib or placebo treatment. The palbociclib group received 125 mg given once daily given from days 1 to 21, followed by a 7-day break in a 28-day cycle of treatment. The placebo group also followed this dosing schedule. Both groups were also given standard neoadjuvant chemotherapy alongside palbociclib or the placebo. The primary outcome of interest was iDFS based on the random assignment of 1250 patients. The end of the trial showed that the use of palbociclib did not improve the iDFS compared to the use of a placebo, ultimately leading to the conclusion that the use of palbociclib for 1 year alongside estrogen therapy did not lead to improvement in iDSF [60]. The primary endpoint, of improving invasive disease-free survival (iDFS) in ERα+, HER2^−^ breast cancer patients who had residual invasive disease after completing neoadjuvant chemotherapy (the phase 3 PENELOPE-B) was not met.

**Ribociclib**. Ribociclib, the second CDK4/6I to become FDA approved, is structurally and functionally similar to palbociclib, and can be used in premenopausal, perimenopausal or postmenopausal women. It is most effective when combined with aromatase inhibitors, including anastrozole, exemestane, and letrozole. Like palbociclib, ribociclib requires fulvestrant adjunct therapy to suppress ovarian function, particularly in postmenopausal patients without prior endocrine therapy [40,70,71,72,73,74]. The most concerning adverse effect of ribociclib use is cardiotoxicity, monitored with electrocardiograms (EKGs).

The MONALEESA trial was a randomized, double-blinded study using oral dosing of ribociclib [63]. The MONALEESA-2 trial examined the clinical benefits of ribociclib (600 mg and higher) over standard therapy with letrozole in patients with advanced metastatic disease (all subtypes and/or ≥1 lytic bone lesion) [39]. Patients were then stratified by either the presence or absence of visceral malignant advancement.

The MONALEESA-3 study was a randomized, placebo-controlled phase III study designed to assess the benefits of ribociclib in combination with fulvestrant for patients with confirmed HR^+^/HER2^−^ ABC, including men and postmenopausal women and patients with advanced metastatic disease (all subtypes and/or ≥1 lytic bone lesion). Combination therapy of ribociclib plus fulvestrant significantly prolonged PFS and OS compared to placebo plus fulvestrant, (Table 2 [64]). The MONALEESA-7 trial extended these findings to examine the effect of ribociclib on PFS rates in comparison to endocrine therapy alone in premenopausal or perimenopausal patients with HR^+^/HER2^−^ ABC [63].

Although CDKIs have been shown to have the best results in conjunction with endocrine therapy, palbociclib and ribociclib in particular, synergize well in combination with phosphoinositide 3-kinase (PI3K) and mammalian target of rapamycin (mTOR) inhibitors. Similar to the effects of CDKIs, antagonism of protein kinase B (AKT) and 3-phosphoinositide-dependent protein kinase 1 (PDK1) produce a similar reduction in tumor growth and progression via cancer cell senescence. Moreover, pairing ribociclib with PI3K/mTOR inhibitors reduced ribociclib resistance, especially in ER^+^/HER2^−^ breast cancer patients, which is mediated by the PI3K/AKT pathway [75].

**Abemaciclib.** A third FDA-approved CDK4/6I for breast cancer management is abemaciclib. Abemaciclib has different clinical features than other CDKIs, presumably due to its more significant inhibition of CDK4 than CDK6 and inhibition of CDK9 [76]. Administration of abemaciclib is provided continuously daily if well tolerated (a twice-daily regimen is permitted), which differs from the dosing schedule for other CDKIs [41,42].

The FDA approved abemaciclib for use in HR^+^/HER2^−^ ABC treatment in 2017 as either monotherapy in patients receiving endocrine therapy or chemotherapy or used in conjunction with fulvestrant in those with prior exposure to hormonal therapy. The MONARCH trials were initiated to investigate abemaciclib therapy’s results in different potential breast cancer management regimens. MONARCH-1, a phase II research trial, examined abemaciclib monotherapy (200 mg twice daily) in HR^+^/HER2^−^ ABC patients with previously significant exposure to treatment with endocrine therapy and chemotherapy [42].

The MONARCH-2 phase III trial randomized patients with HR^+^/HER2^−^ ABC to either fulvestrant plus abemaciclib (150 mg twice daily) or placebo [66]. PFS and OS of the combined therapy group was significantly greater than placebo (Table 2). In addition, the combination of fulvestrant with abemaciclib conveyed beneficial results in patients with primary endocrine therapy resistance, significantly increasing overall survival [66]. The MONARCH-3 trial demonstrated similar effectiveness of combination abemaciclib therapy with aromatase inhibitors in the same patient demographic as MONARCH-2 [68].

The MONARCH trial indicated the use of abemaciclib with aromatase inhibitors for postmenopausal women without prior hormonal therapy exposure. There was also robust evidence for combining abemaciclib with fulvestrant to suppress ovarian functions in premenopausal and perimenopausal women. Lastly, there was supporting evidence that both male and female patients should receive abemaciclib as monotherapy after ineffective use of anti-hormonal and chemotherapy for the management of metastatic breast cancer [67].

Further differences between abemaciclib and other CDKIs result from its capacity to cross the blood–brain barrier (BBB), thus allowing penetration into the cerebrospinal fluid (CSF). This characteristic is essential for the treatment of brain metastasis in breast cancer patients [77,78,79,80,81,82]. Abemaciclib’s ability to adequately reach the central nervous system results in substantial improvement in morbidity of metastatic breast cancer patients and potentially for other primary CNS malignancies. The ability of CDKIs to accumulate in CSF depends on many factors, particularly the presence of p-glycoprotein (P-gp) and breast cancer resistance proteins in the CNS. These proteins act to extract and dispose of certain drugs from the brain, including palbociclib and abemaciclib [77]. Although both these drugs can penetrate the BBB, they display different levels of accumulated CSF concentrations due to palbociclib’s greater efflux by P-gp. Thus, abemaciclib is the most effective CDKI to manage brain metastasis of breast malignancies [41]. Pharmacodynamic markers are used to monitor patient response to abemaciclib therapy to observe treatment efficacy. These markers include the expected decline in phosphorylated-Rb protein and topoisomerase II-alpha, which are associated with treatment competence [41].

## 5. Dose-Limiting Side Effects

Although each CDKI can produce side effects, the most common ones seen with these drugs include bone marrow suppression, cardiotoxicity, hepatotoxicity, and gastric toxicity, and most are contraindicated in pregnant patients. Common side effects of CDKIs include pancytopenia, particularly neutropenia, lethargy, fatigue, nausea, diarrhea, and low-grade alopecia. The high rates of febrile neutropenia are monitored by complete blood counts (CBCs) with differential. These effects are generally resolved after discontinuation of CDKI therapy [34,35,36].

The PALOMA trials highlighted side effects and precise dosing as essential in obtaining the most therapeutic value with palbociclib. The most significant side effect of palbociclib is acute neutropenia (up to 62% of patients), resulting in immunosuppression and infections managed with antibiotics [83]. The time taken for the appearance of neutropenia from initiation of treatment was 20 days, with the recurrence slowly reducing over therapy use [37]. Monitoring for adverse effects via CBCs with differential is conducted regularly, recommended on day 14 and performed if any complications arise within the treatment period [34,35,36].

Toxic effects of ribociclib are similar to those of palbociclib, with the addition of cardiotoxic side effects with ribociclib. These effects are monitored with routine EKGs. Dose-dependent prolongation of the QT interval is seen at a dose of 600 mg. Detection of QT interval with Fridericia’s correction (QTcF) was observed to be >480 milliseconds (ms), whereas the standard length of this period is ideally 400–440 ms. This conduction abnormality can lead to irregular heartbeats and eventually life-threatening arrhythmias. As such, patients with prior cardiac disease, prolonged QTcF or those taking any other medications that increase the risk of QT lengthening are not approved to be involved in this study (30). Routine monitoring of hepatic transaminases with liver function tests (LFTs) and bilirubin levels is required to prevent liver complications. These effects are generally resolved after discontinuation of ribociclib (30).

A significant distinction between abemaciclib and other CDKIs is its minimal hematologic adverse effects, with mild leukopenia seen in patients rather than the expected high-grade neutropenia [4,41,42]. The more common and more favorable side effects seen with abemaciclib use include fatigue and gastrointestinal toxicity, particularly abdominal pain, diarrhea, and nausea [66]. These may lead to dehydration and infection; however, management with Imodium (loperamide), greater fluid intake, and changes in diet can relieve these symptoms. Abemaciclib is not recommended in patients with any underlying gastrointestinal conditions such as irritable bowel syndrome, colitis, or diverticulitis [84,85]. Additionally, an observed elevation of creatinine is seen with abemaciclib use; however, it may not be directly associated with renal impairment but due to drug interference with creatinine tubular secretion [41]. These benign adverse effects are managed with dose reductions or modifications, particularly during the first two months of initial therapy. Intolerance to side effects may require a break from treatment, as with any serious complications [86].

The dosage for palbociclib and ribociclib administration follows the same schedule, with intake for three weeks (21 days) followed by a one-week (7-day) interval without CDKI use to complete a 28-day cycle. However, letrozole (2.5 mg) is taken continuously throughout the four weeks of the combination drug regimen. Initial palbociclib dosing begins at 125 mg, and ribociclib is dosed at 600 mg (dispensed as three 200 mg tablets). Fulvestrant administration is given intramuscularly (500 mg) on the first day of every 28-day cycle, administered as two injections in each gluteal muscle. Additionally, in the first cycle, an extra fulvestrant dose is provided for day 15 [63,64,87]. Abemaciclib administration is provided continuously daily, permitting a 150 to 200 mg dosing regimen every 12 h (twice daily) if well tolerated [41,42].

## 6. Mechanisms of Resistance to CDK4/6 Inhibitors

A review of the clinical trials (Table 2) illustrates the vital impact the new generation of CDK inhibitors has had, yet raises several important questions. Firstly, CDK activity remains inhibited in the patients’ tumors, yet the tumors progress. The reduction in RB phosphorylation as the tumor progress suggests CDK independent pathways maintain tumor growth. The development of resistance to CDK4/6I is typical in most patients. The tumors being treated have high levels of chromosomal instability, and consequent genomic instability [29] as cyclin D1 overexpression is a driver of chromosomal instability [29,88,89]. Consistent with the high level of CIN, genomic alterations occur with CDKI therapy, including loss of the RB gene, altered cyclin E1 expression [90], p27 inhibition [91], and activation of the Phosphatidylinositol-4,5-Bisphosphate 3-Kinase Catalytic Subunit Alpha (PIK3CA) pathway [92].

**Genetic loss and mutations of RB**. RB is a tumor suppressor protein that acts as a critical checkpoint regulator of the G1/S phase of the cell cycle and is, therefore the main target of CDK4/6Is to cause cell-autonomous G1 arrest. Rb regulates E2F, a downstream transcription factor. E2F bound to Rb restrains induction of the G1 to S phase cell cycle. RB genetic loss or silencing causes CDKI resistance [93,94,95,96]. Loss of RB is associated with increased E2F expression leading to constitutive activation of downstream proteins. E2F can upregulate AKT signaling via GRB2 Associated Binding Protein 2 (Gab2) [97]. In tumors where Rb is inactivated, the targeted inhibition of the cyclin E-CDK2 axis in combination with CDK4/6Is may effectively overcome resistance to CDK4/6Is [54,98].

**Cyclin E1 and cyclin E2 as biomarkers of response to CDK4/6 inhibitors.** Cyclin E1 overexpression may constitute a resistance mechanism in patients treated with fulvestrant + palbociclib. CDK4/6 inhibitor resistance is associated with increased CDK2 activation (reviewed in [99]. In such circumstances, CDK2/cyclin E inhibitors such as Cyclacel (CYC065) may be of use [100]. The PALOMA-2 and PALOMA-3 trials showed that palbociclib addition to endocrine therapy benefited recurrence-free survival irrespective of *CCNE1* and *CCNE2* levels in the pre-treated primary tumor samples. However, the expression of *CCNE1* distinguished patients with the longest vs. the shortest recurrence-free survival. Cyclin E1 is not amplified in ERα^+^ breast cancer, nor does amplification appear to occur during progression to resistance [101]. In both the MONALEESA-2 (letrozole/ribociclib) and the PALOMA-3 (fulvestrant/palbociclib) trials, high expression of *CCNE1* mRNA was associated with poor progression-free survival [100]. In the PALOMA-3 trial, high pre-existing *CCNE2* mRNA expression was not associated with any difference in progression-free survival [101], nor did *CCNE2* amplification increase during disease progression [102]. In the MONALEESA-7 and CLEE011X2106 trials there was a trend for *CCNE1*-high patients to have poor progression-free survival. Furthermore, patients expressing cytoplasmic cyclin E1 protein, reflecting a low molecular weight form of cyclin E1, had worse survival than patients expressing only nuclear cyclin E1 protein [103]. In the POP trial of pre-operative palbociclib, *CCNE2* expression was significantly decreased in antiproliferative responders vs. non-responders measured over 15 days (*p*  =  0.006) [104]. *CCNE1* was high in patients who maintained high Ki67 after 15 days of treatment in the NeoPalAna study [44] in the PALOMA-3 trial [102].

**p16 Amplification**. The tumor suppressor p16^INK4a^, a member of the inhibitors of the CDK4 (INK4) family and a natural inhibitor of CDK4, is involved in cell cycle control and regulation [92]. Since CDK4/6 (a p16 target) requires Rb for its kinase activity, p16 acts as a tumor suppressor when functional Rb is present [105]. p16 overexpression occurs during oncogenic stress in the presence or absence of Rb [106,107]. In human breast cancer, p16^INK4a^ is inversely correlated with cyclin D1 and ERα expression [108]. p16 expression lacked prognostic relevance in TNBC [109]. p16^INK4a^ inactivation by DNA methylation occurs in ≤30% of human breast cancers [110] however increased abundance is also reported [109]. In mouse models of tumorigenesis *Ink4a/Arf*^+/−^ mice have increased *Eμ-Myc*-induced lymphomagenesis and epidermal growth factor receptor-induced gliomagenesis. In ErbB2-induced mammary tumorigenesis, *Ink4a/Arf*^+/−^ mice showed decreased p16^INK4a^, increased Ki-67 expression, increased cyclin D1 protein but decreased mammary tumor apoptosis [111]. Currently, two theories exist involving loss of Rb and amplification of p16 [112]. Further studies might be beneficial in designing the strategies to overcome acquired resistance to CDK4/6Is.

**Figure 4:**
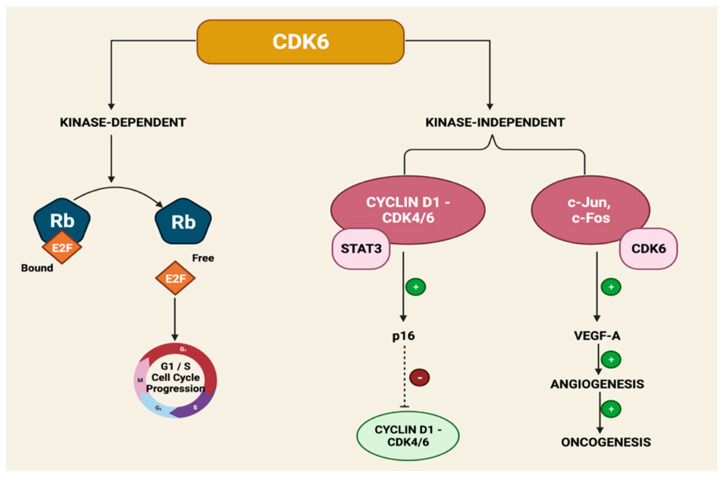
Mechanism of CDK6 Amplification in Resistance to CDK4/6 Inhibitors.

**CDK6 Amplification**. The catalytic subunit CDK6 conveys both kinase-dependent and kinase-independent roles via gene transcription [113]. CDK6 upregulates p16INK4a in the presence of STAT3 and cyclin D1 [114]. CDK6 increases vascular endothelial growth factor A (VEGF-A) and c-Jun, promoting angiogenesis to allow the promotion of breast cancer progression and CDK4/6I resistance [115] (Figure 4).

**CDK4 Amplification.** CDK4 can be amplified via gene amplification, mutations, and epigenetic alterations, which allows overactivation of the cyclin D1-CDK4/6-Rb pathways. Although CDK4 overexpression has been seen in several cancers and may limit the therapeutic efficacy of CDK4/6Is [116,117], this is an uncommon event in breast cancer.

**Activation of the FGFR Pathway.** The fibroblast growth factor receptor (FGFR) leads to a signaling pathway that plays a vital role in proliferation, differentiation, and cell survival [118]. The FGFR pathways, particularly FGFR1-4, are overactivated in breast cancer and other malignancies and are implicated in cancer progression [119]. Therefore, FGFR1 and FGFR2 are associated with CDK4/6I resistance development and endocrine therapy resistance. FGFR1 amplification activates of the PI3K/AKT and RAS/MEK/ERK signaling pathways, specifically in endocrine-resistant breast cancer cells [120,121]. Laboratory-induced FGFR1 overexpression showed resistance to combination therapy with palbociclib/fulvestrant and fulvestrant monotherapy [122]. FGFR2 is the main activating factor of the FGFR pathway and functions to promote endocrine resistance. Therefore, a potential option in overcoming CDKI resistance may require combined inhibition of both CDK4/6 and FGFR pathways by combining brivanib, an FGFR-1/VEGFR-2 kinase inhibitor, with tamoxifen, which could potentially maximize the therapeutic efficacy and rescue cells’ sensitivity to endocrine therapy [121].

**Activation of the PI3K/AKT/mTOR Pathway**. In approximately 30–40% of breast cancer cases, especially HR^+^ subtypes, activation of the PI3K/AKT/mTOR signaling pathways are seen [93,122]. Dysregulation of this pathway can lead to critical resistance to endocrine therapy and was recently reported to be associated with CDK4/6I resistance [75]. Patients with CDKI-resistant breast cancer cells have become more dependent on PI3K/AKT/mTOR signaling pathways rather than ERα signaling for oncogenesis. CDK4/6I phosphorylate AKT via PDK1 to activate PI3K/AKT/mTOR pathways in ribociclib-resistant breast cancer cells. CDK4/6I-resistant breast cancer cell lines show reactivation of phosphorylated-Rb and E2F, which may occur via the CDK or the mTOR pathways [123]. A recent study suggested that PI3K inhibitors may downregulate cyclin D1 expression and promote resistance to CDK4/6Is. Therefore, there are potential therapeutic benefits in combination therapy of PI3K/AKT/mTOR inhibitors and CDK4/6Is in addressing CDKI resistance and enhancing the anticancer effect in CDKI-sensitive cases [93].

**Loss of ERα or PR Expression**. A critical factor in the progression of breast carcinogenesis is cyclin D1-CDK4/6 activity which allows hormone-mediated activation of ER [37]. Abemaciclib-resistant breast cancer cell lines exhibited loss of ER/PR expression [115]. This results in the loss of the estrogen-dependent driver of tumor growth; however, this can also lead to a mechanism of resistance to CDK4/6I therapy. In addition, patients who develop resistance mechanisms to CDKI treatment may require treatment methods beneficial in HR^−^ subtypes of breast cancers.

**Higher transcriptional activity of AP-1 transcription factor**. The structure of activator protein 1 (AP-1) is a heterodimer composed of proteins belonging to the c-Fos, c-Jun, activation transcription factor (ATF), and transcription factor MAF sub-families [124]. Approximately 20–40% of breast cancers have increased c-Jun activity [125]. Selective c-*jun* gene deletion in mice evidenced a role for c-Jun in maintaining breast epithelial cell survival [126]. Laser capture microdissection demonstrated endogenous c-Jun inhibited expression of apoptosis-inducing genes and reactive oxygen species (ROS)-reducing genes (MnSOD, catalase) [126]. In breast tumors c-Jun is expressed at the invading edge [127], suggesting a role for c-Jun in tumor migration. Consistent with this clinical observation, somatic deletion of the c-*jun* gene, conducted using floxed c-*jun* (c-*jun^f/f^*) conditional knockout mice, showed c-Jun promoted cellular migration and invasion via increasing expression of CCL5 [128] and stem cell factor (SCF) [129], increasing c-Src abundance and suppressing Rock kinase signaling [130]. Increased transcriptional activity of AP-1 and increased c-Fos levels were noted to lead to acquired resistance to palbociclib and tamoxifen (96). Inhibiting AP-1 in combination with palbociclib and fulvestrant was ultimately more productive dual or mono-treatment (96). One c-Fos/AP-1 inhibitor (T-5224) has reached Stage II of trials [124].

**Immune Mechanisms of CDKI therapy resistance**. CDKi have significant and diverse effects on the breast cancer tumor immune microenvironment (reviewed in: [2]). Stromal cyclin D1 is increased in human breast cancer, correlating with poor outcome, and is known to augment the recruitment of macrophages into the breast cancer tumor microenvironment [131]. Treatment with immune checkpoint inhibitors is being explored for TNBC [132]. Compared with HER2-positive breast cancer and TNBC, ERα-positive breast cancer is not an immunogenic cancer type. Luminal breast cancers have the lowest level of tumor PD-L1 expression compared with basal-like and HER2-positive tumors. However, CDK4/6I-resistant breast cancer cells demonstrated upregulation of IFN-ɑ and IFN-β activity in immune-related signaling pathways [125,133]. DNA methyltransferase is an E2F target protein that promotes cytotoxic T-cell-mediated tumor inhibition when CDK4/6Is inhibit its activity [134]. CDK6 phosphorylates and thereby inhibits the nuclear factor of activated T-cell 4 (NFAT4), [135], reducing interleukin 2 (IL-2) levels. CDK4/6Is dephosphorylate NFAT4, enhancing its activity and increasing IL-2 levels [135]. CDKIs can potentiate anti-tumor immunity via augmenting the response to programmed cell death protein 1 (PD-1) blockade. Combining therapy with CDK4/6Is and PD-1 inhibitors may be a useful approach to overcome CDK4/6Is resistance.

## 7. Identifying Targeted Therapies for CDK4/6 Inhibitor-Resistant ER+ Breast Cancer

It has been proposed CDKI resistant cells have increased dependency upon the G2/M checkpoint [136]. WEE1 is an important G2 checkpoint regulator. In ER+ breast cancer cell models made resistant to ribociclib, the combination of AZD1775 (WEE1 inhibitor) and ribociclib inhibited proliferation in resistant cells [136]. Inhibiting WEE1 kinase decreased cell proliferation and increased G2/M arrest, apoptosis, and gamma H2AX levels (a marker for DNA double-strand breaks), in resistant cells compared with sensitive cells [136]. WEE1 kinase is thus a promising anticancer target in therapy-resistant ERα+ breast cancer [136].

## 8. Conclusions

Breast cancers, particularly metastatic HR^+^/HER2^−^ advanced breast carcinomas, are currently treated with CDK4/6I [39,55,56]. However, tumor progression occurs frequently despite CDKi therapy. Recent studies, including the phase 3 PENELOPE-B trial, which did not meet the primary endpoint of improved invasive disease-free survival (iDFS) suggest there is much more to be understood about the role of CDK in breast cancer.

The variety of distinct genetic mechanisms giving rise to therapy resistance may, in part, reflect the intrinsic genomic instability of cyclin D1 overexpressing breast tumors [88] (Figure 3A). Cyclin D1 induced chromosomal instability (CIN) in murine mammary epithelium [29,88], and in other species [89]. Cyclin D1 is induced early in breast cancer and CIN is an early feature of tumorigenesis that may precede tumor suppressor loss [137,138]. Cyclin D1 [88] and cyclin E, are both capable of inducing CIN [139], and the induction of cyclin E in CDKI-resistant tumors may sustain CIN. Cyclin D1 induction of CIN is in mouse hepatocytes [140], lymphoid tumors [141] and bladder cancer [142]. The recent identification of drugs targeting CIN [143,144] may provide a rational basis for therapeutic substratification, supplementing with compounds targeting CIN in the luminal B subtype of breast cancer.

Multiple cell-cycle states have cell-cycle states have specific genetic and pharmaceutical vulnerabilities and a high degree of heterogeneity within tumor types [145]. RB loss for example, results in specific new vulnerabilities for therapeutic intervention, such as the use of the XIAP/CIAP inhibitor birinapant [146]. Furthermore, the combination of AURK and WEE1 inhibitors, yields synergistic cell death selectively in RB-deleted ERα^+^ breast cancer cells. WEE1 is targeted to inhibit the growth of breast cancer cells resistant to endocrine therapy and CDK4/6 inhibitors [136]. Although remarkable progress has been made in understanding the utility of CDKI for breast cancer,

## Figures and Tables

**Table 1:** Summary of FDA approved CDK4/6 Inhibitors.

Drug	Mechanism of Action IC50 (nM)	Recommended Dose Half-Life Tmax	Drug Interactions IC50 against Bone Marrow Mononuclear Cells (nM)	References
**Palbociclib**	Similar potency against cyclin D1/CDK4 and cyclin D2/CDK6	125 mg po once a day for 21 days in a 28-day cycle with food	CYP3A4 substrate 240 ± 43	[34,35,36,37,38]
CDK4–cyclin D1	11			
CDK6–cyclin D1/2/3	16	Half-life		
CDK1–cyclin B	>10,000	26–27 h		
CDK2–cyclin A/E	>10,000	Tmax		
CDK5–p25	>10,000	6–12 h		
**Ribociclib**	Greater potency against CDK4 than CDK 6	600 mg po once daily for 21 days in a 28- day cycle with or without food	CYP3A4 substrate 1700 ± 231	[39,40]
CDK4–cyclin D1	10			
CDK6–cyclin D1/2/3	39	Half-life		
CDK1–cyclin B	113,000	33–42 h		
CDK2–cyclin A/E	76,000	Tmax		
CDK5–p25	43,900	1–5 h		
**Abemaciclib**	Greater potency against CDK4 than CDK 6 (CDK4 and CDK6 with IC50 of 2 nM and 10 nM	150 mg or 200 mg po BID with or without food.	CYP3A4 substrate, BCRP, Pgp 230 ± 27	[4,41,42,43]
CDK4–cyclin D1	2			
CDK6–cyclin D1/2/3	10	Half-life		
CDK1–cyclin B	1627	17–38 h		
CDK2–cyclin A/E	504	Tmax		
CDK5–p25	355	4–6 h		

**Table 2:** Summary of CDK4/6 Inhibitors and Corresponding Research Trials.

DRUG	TRIAL	TARGET POPULATION	EXPERIMENTALGROUP	CONTROL GROUP	CHANGE IN PFS	CHANGE IN OS
**PALBOCICLIB**	PALOMA-1	165 postmenopausal	+Letrozole	Letrozole monotherapy	20.2 vs. 12.9 months [34,35,53,54,61]	[34,35,53,54,61]
	PALOMA-2	666 postmenopausal		Placebo + Letrozole	24.8 vs. 14.5 months [34,35,53,61]	
	PALOMA-3	521 pre/peri/post-menopausal	+Fulvestrant	Placebo + Fulvestrant	9.2 vs. 3.8 months [34,35,53,57,61]	6 years OS, 19.1% vs. 12.9% [62]
	PALLAS	5796 (5761 included in analysis)	+Endocrine adjuvant	Endocrine adjuvant	N/A [34,35,53,59]	
	PENELOPE-B	1250 included in analysis	+standard endocrine adjuvant	Placebo + standard endocrine adjuvant	N/A [60]	
**RIBOCICLIB**	MONALEESA-2	668 postmenopausal	+Letrozole	Placebo + Letrozole	20.5 vs. 12.8 months [53,61,63,64] [53,63,64]	
	MONALEESA-3	726 men and postmenopausal women with prior exposure to ET	+Fulvestrant	Placebo + Fulvestrant		42 months OS 57.8% vs. 45.9% [65]
	MONALEESA-7	672 pre/peri-menopausal	+Fulvestrant + Goserelin	ET + Goserelin	23.8 vs. 13.0 months [53,61,63,64]	
**ABEMACICLIB**	MONARCH-1	132 with prior ET or chemo exposure	Monotherapy	N/A	16.4 vs. 9.3 months [42,53,61] [53,66]	
	MONARCH-2	669 with prior ET exposure	+Fulvestrant	Placebo + Fulvestrant		46.7 months OS vs. 37.3 months [67]
	MONARCH-3	493 postmenopausal	+Letrozole	Placebo + Letrozole	28.2 vs. 14.8 months [53,61,68]	

* ET = endocrine therapy [69]. N/A primary end point not achieved.
